# One mechanism of Sishen Pill on diarrhea with kidney Yang deficiency syndrome: influencing metabolic function by intestinal microorganisms and enzyme activity mediates the gut-kidney axis

**DOI:** 10.3389/fcimb.2025.1620789

**Published:** 2025-08-14

**Authors:** Mengsi Zhou, Xiaoya Li, Nenqun Xiao, Zhoujin Tan

**Affiliations:** ^1^ School of Pharmacy, Hunan University of Chinese Medicine, Changsha, Hunan, China; ^2^ Hunan Key Laboratory of Traditional Chinese Medicine Prescription and Syndromes Translational Medicine, Hunan University of Chinese Medicine, Changsha, China; ^3^ School of Chinese Medicine, Hunan University of Chinese Medicine, Changsha, Hunan, China; ^4^ Yunnan Provincial Key Laboratory of Molecular Biology for Sinomedicine, Yunnan University of Traditional Chinese Medicine, Kunming, Yunnan, China

**Keywords:** Sishen Pill, diarrhea with kidney-Yang deficiency syndrome, intestinal microorganisms, enzyme activity, microbial activity, gut-kidney axis

## Abstract

**Introduction:**

Sishen Pill (SSP), a classic TCM formula, warms the kidney and spleen, astringes the intestine, and stops diarrhea. Emerging evidence suggests that diarrhea with KYDS is linked to gut microbiota imbalance and altered intestinal enzyme activities. At the same time, SSP has been shown to regulate gut microbiota, improve metabolism, and alleviate intestinal disorders. This research investigates how SSP prevents and treats diarrhea by studying the interaction between SSP and intestinal microorganisms.

**Methods:**

In a murine model of diarrhea induced by adenine and *Foliuem sennae* co-administration, we collected various biospecimens, including intestinal mucosa (ileum and colon), luminal contents, serum, and major organs (kidney, spleen) for comprehensive mechanistic analyses. Techniques such as microbial culture, enzyme activity assays, and HE staining were employed to assess cultivable microbial colony counts, enzyme activity, relevant metabolic indicators, oxidative stress markers, and to observe kidney tissue sections.

**Results:**

The results indicated that SSP treatment significantly reduced uric acid levels, Escherichia coli (E. coli) count, and amylase activity compared to the spontaneous recovery (MC) group, while the spleen and thymus index, total bacterial count, sucrase activity in contents, protease activity and microbial activity in mucosa were significantly higher than the measurements in MC group. Significant differences were observed in alanine aminotransferase level, Lactobacillus count, Bifidobacterium count, sucrase activity, and microbial activity between the SSP and blank control groups. Serum uric acid levels showed a positive correlation with E. coli colony count and a negative correlation with Lactobacillus colony count. Additionally, total bacterial colony count was negatively correlated with aspartate aminotransferase levels.

**Conclusions:**

The SSP may alleviate diarrhea with kidney Yang deficiency syndrome by reducing E. coli count, enhancing specific enzyme activities, and regulating organ indices and oxidative stress, with the regulatory effects on organ indices and oxidative stress potentially associated with its modulation of E. coli and enzyme activity. This cascade of microbial-enzymatic regulation likely contributes to the normalization of organ indices (e.g., spleen and thymus indices) and alleviation of oxidative stress, as reflected by enhanced superoxide dismutase activity. These findings highlight the multitarget therapeutic potential of SSP in addressing dysfunction in the intestinal-microbiome-enzymatic-organ axis in diarrhea with kidney Yang deficiency syndrome.

## Graphical Abstract

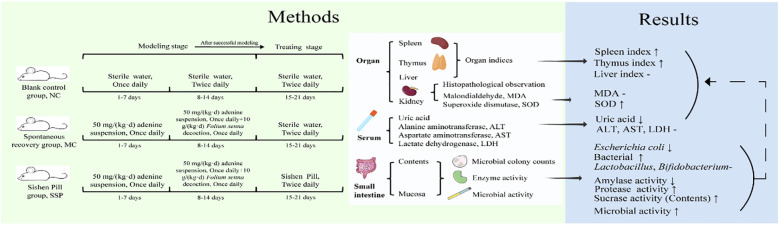

## 1 Introduction

Diarrhea refers to the frequent passage of loose stools, possibly containing undigested food, or watery stools in severe cases. The primary pathogenesis of diarrhea is Spleen deficiency with dampness, which is closely associated with the functions of the intestines, liver, and kidneys (Wang et al., 2017). In the theory of traditional Chinese medicine (TCM), the concept of zangfu extends beyond a mere anatomical description, representing a comprehensive functional system that integrates physiological function and pathological manifestations. In this context, the connotation of Pi (脾, spleen) encompasses both anatomical and functional aspects, which are interrelated and inseparable. The primary function of the Pi is summarized as “the spleen governs transportation and transformation” and “the spleen holds blood”. Anatomically, the spleen and pancreas, along with the stomach, large intestine, and small intestine, form the digestive and transport system, with the spleen serving as the core (Liu et al., 2023). The Shen (肾, kidney) in TCM refers to a functional system centered on the kidney, encompassing various tissues and organs, including the kidney, bladder, bone, and marrow. Through the Qi transformation of Kidney essence, it shows two functions, kidney Yin and kidney Yang, so as to adjust the relationship between each system (Liu et al., 2024). Long-term diarrhea cannot be cured, the disease is changed from excess pattern to deficiency pattern, spleen deficiency leads to kidney deficiency, or kidney Yang deficiency, cannot warm the spleen Yang, help the spleen transportation and transformation, indigestion of food, and eventually develop into diarrhea with kidney Yang deficiency syndrome (KYDS).

Sishen Pill (SSP) was first recorded in the *Huatuo Shenyi Mizhuan* (华佗神医秘传, Secret Biography of Hua Tuo Miracle Doctor, Tang Dynasty), compiled by Simiao Sun under the name of *Huatuo Zhi Shenxie Shenfang* (华佗治肾泄神方, *Huatuo’s divine formula for treating kidney diarrhea*). Later, it was collected as “Sishen Pill” in *Chenshi Xiao’er Douzhen Fanglun* (陈氏小儿痘疹方论, Chen’s Pediatric Chickenpox and Rash Prescription Theory, Song Dynasty). Volume 6 of *Zhengzhi Zhunsheng: Lei Fang* (证治准绳•类方, Zhengzhi Zhunsheng-Category Formulas, Ming Dynasty) defined the dosage of each Chinese herbal medicine of SSP, so later generations of doctors believed that SSP came from this literature, and later generations of prescription books also referred to this literature to determine the dosage of each Chinese herbal medicine of SSP (Gao and Li, 2011). SSP comprises *Bu Gu Zhi* (*Psoralea corylifolia* L.), *Rou Dou Kou* (*Myristica fragrans* Houtt.), *Wu Zhu Yu* (*Tetradium ruticarpum* (A.Juss.) T.G.Hartley), *Wu Wei Zi* (*Schisandra chinensis* (Turcz.) Baill.), *Sheng Jiang* (*Zingiber officinale* Rosc.), and *Da Zao* (*Ziziphus jujuba* Mill.). In the compound formulas, *Bu Gu Zhi* is the monarch medicine to warm and tonify the kidney Yang, and *Rou Dou Kou* is the minister medicine to warm and tonify the spleen Yang and stomach Yang, and astringent intestine to stop diarrhea. Combined with *Wu Zhu Yu*, it can warm the spleen and kidney to disperse the cold. The flavor of *Wu Wei Zi* is sweet and acidic, warm, into the kidney meridian, warm kidney Qi, and astringent. *Sheng Jiang* warms the stomach and dissipates cold, and *Da Zao* tonifies the spleen and nourishes the stomach, both as guide medicine. Combining six Chinese herbal medicines can warm the kidney and spleen, astringe the intestine, and stop diarrhea (Wei et al., 2021).

The occurrence and development of diarrhea with KYDS are closely linked to gut microbiota. Previous studies have found that the number of *Lactobacillus* and *Bifidobacterium* colonies in the intestinal contents of mice with diarrhea with KYDS is significantly reduced, and the number of *E. coli* colonies is significantly increased. The activities of lactase, amylase and sucrase in the intestinal mucosa are significantly increased, whereas the activity of xylanase in both the intestinal contents and mucosa is significantly decreased. Furthermore, microbial activity in the intestinal mucosa is notably reduced (Zhou et al., 2024b). Based on 16S rRNA High-Throughput Sequencing, 50 mg/(kg·d) adenine suspension combined with 10 g/(kg·d) *Foliuem sennae* decoction can cause diarrhea in mice, and change the structure and function of gut content microbiota in mice, resulting in the enrichment of characteristic bacteria *Lactobacillus intestinalis* and *Bacteroides acidifaciens* (Li et al., 2022a). Previous studies have found that SSP can reduce CD11c^+^CD103^+^E-cadherin^+^ cells and proinflammatory cytokines levels, regulating gut microbiota (beneficial bacteria are increased and pathogenic bacteria are reduced) to treat DSS-induced inflammatory bowel disease (Chen et al., 2020). SSP restores gut microbiota imbalance and improves fecal metabolic dysfunction in mice with colitis with spleen-kidney Yang deficiency.

Correlation analyses reveal that SSP’s modulatory effects on free triiodothyronine, free thyroxine, interleukin-10, colonic weight index, and immune cell subsets (CD103^+^CD11c^+^TNF-α^+^ and CD103^+^CD11c^+^MHC-II^+^), along with 13 differentially expressed metabolites, are significantly associated with altered abundances of specific gut microbiota taxa, including *Parvibacter*, *Aerococcus*, *norank f Lachnospiraceae*, *Lachnospiraceae* UCG-006, *Akkermansia*, and *Rhodococcus* (Ge et al., 2022). SSP alleviates ulcerative colitis progression by increasing gut microbiota diversity and butyrate levels, thereby restoring the Treg/Th17 immune balance and intestinal homeostasis (Wang et al., 2022c). Notably, findings demonstrate that SSP alleviates diarrhea with KYDS by modulating the *Lactobacillus johnsonii*-propionic acid metabolic pathway and improving energy metabolism and the diversity and structure of mucosa-associated gut microbiota (Li et al., 2024; Zhu et al., 2023). In addition, SSP can promote the production of the intestinal metabolite short-chain fatty acids, reduce intestinal injury, maintain the integrity of the intestinal mucosal barrier, regulate signaling pathways, regulate intestinal epithelial cytokines, and regulate the balance of immune cells. It has a good effect on digestive system diseases such as inflammatory bowel disease and diarrhea-type irritable bowel syndrome (Zhang et al., 2021; Liu et al., 2020; Wang et al., 2022b; Lin et al., 2020).

Therefore, we hypothesize that SSP can ameliorate diarrhea with KYDS by modulating intestinal microorganisms and enzyme activity. In this study, we investigate the effects of SSP on intestinal microorganisms, enzyme activity, and oxidative stress in mice with diarrhea with KYDS using techniques such as microbial culture, enzyme activity assays, and biochemical analysis. Our goal is to elucidate the underlying mechanism of SSP in treating diarrhea with KYDS from the perspective of intestinal microorganisms and enzyme activity, and to provide novel insights into its mechanism of action and potential therapeutic targets.

## 2 Materials and methods

### 2.1 Preparation of medicine

Adenine suspension: Weigh an appropriate amount of adenine (Changsha Yaer Biotechnology Co., Ltd., batch number: EZ2811A135) every day according to the weight of the mouse, and dissolve it in sterile water, and arrange it into a 50 mg/(kg·d) adenine suspension (Zhou et al., 2024a).

*Foliuem sennae* decoction: Take the washed *Foliuem sennae* (Anhui Puren TCM Decoction Decoction-ready medicines Co., LTD., batch number: 2005302), soak in 75-80°C hot water for 15 min with 10 times the amount of medicinal materials, filter through gauze, concentrate to 1 g/mL by rotary evaporator, and store at 4°C for further use.

Composition and dosage of SSP: *Bu Gu Zhi* (Salt-fried, 12 g), *Rou Dou Kou* (Roasted in ashes, 6 g), *Wu Zhu Yu* (Stir-fried by dipping, 3 g), *Wu Wei Zi* (Vinegar-steamed, 6 g), *Sheng Jiang* (6 g), *Da Zao* (6* g*). According to the conversion method in the conversion of experimental animal and human drug dosage in the *Methodology of Pharmacological Research in Traditional Chinese Medicine* (Chen, 1993), the equivalent dosage of SSP was determined to be 5 g/(kg·d). TCM decoction-ready medicines are provided by Hunan Junhao TCM Decoction Pieces Science and Trade Co., LTD. Batch numbers were HY21012201, Xiang 20160111, 2020082804, HY21020304, 2103120082, 170903.

Preparation of water decoction of SSP: The medicinal materials were weighed according to the proportion of drug use, and 5 times the water was added to the Chinese herbal medicines, soaked for 30 min. After bringing to a boil over high heat, reduce to low heat and simmer for 30 min, filter out the liquid medicine. Add 200 mL of water to the filtered drug residue again, decocting the same method as before, and filter out the drug solution. The liquid medicine was mixed twice and concentrated to 0.29 g crude drug/mL, which was stored at 4°C for later use.

### 2.2 Reagents and kits

References for the preparation of chemical reagents (ONPG, FDA, DNS, etc.) used to detect enzyme activity in intestinal contents (Guo et al., 2024). Malondialdehyde (MDA) kit and Superoxide dismutase (SOD) kit (MDA and SOD were both purchased from Beijing Leagene Biotechnology Co.).

### 2.3 Animals

Thirty male Kunming mice weighing 20 ± 2 g were selected as experimental subjects (All mice were purchased from Hunan Slix Laboratory Co., LTD. Certificate number: ZS-202106150014). To minimize the impact of sex on gut microbiota composition, male mice were consistently chosen (Wu et al., 2022). All mice were housed in a barrier environment, with a temperature of 23-25°C, humidity ranging from 50% to 70%, a 12-hour light/dark cycle, and ad libitum access to food and water. The feed was the growth and reproduction feed for Co^60^ irradiated experimental mice, which was purchased from Jiangsu Medisen Biomedical Co., LTD, batch number SYXK(Xiang)2020-0006. The nutrients mainly included water, crude protein, crude fiber, crude fat, crude ash, calcium, total phosphorus, lysine, methionine, and cystine. The experiment complied with the requirements of the Animal Ethics and Welfare Committee of Hunan University of Chinese Medicine (The ethics approval number: LLBH-202106120002).

### 2.4 Animal grouping, modeling, and treatment protocol

Following a 3-day acclimatization period, thirty mice were randomly allocated into three groups according to the random number table: a blank control (NC) group, a spontaneous recovery (MC) group, and the SSP group (n=10). Modeling stage: Mice in the MC and SSP groups were treated with 50 mg/(kg·d) adenine suspension by gavage, 0.4 mL per administration, administered once daily for 14 days, and 10 g/(kg·d) *Foliuem sennae* decoction, 0.4 mL per administration, administered once daily for 7 days, from the 8^th^ day (Li et al., 2022b). The mice in the NC group received an equivalent volume of sterile water by gavage once daily for 7 days and twice daily from the 8^th^ day onwards. Treating stage: after modeling, the mice in the SSP group were gavaged with 0.4 mL of SSP decoction twice a day for 7 days. The mice in the NC and MC groups were gavaged with an equal volume of sterile water twice a day for 7 days.

### 2.5 Model evaluation criteria

According to the clinical manifestations of diarrhea with KYDS, referring to the “*Model Building and Validation of Diarrhea Mice with Kidney-yang Depletion Syndrome*”, The evaluation criteria for the mouse model of diarrhea with KYDS are as follows: Loose stool, or indigestion of food, cold limbs, emaciation, and listlessness. During the experiment, the behavioral indicators of mice were observed, including mental state, activity frequency, hair shape and color, nail color, feces shape and color, and perianal cleanliness. The body weight, rectal temperature, and diarrhea of mice were detected and recorded. In the experiment, we detected the clinical manifestations of diarrhea index in mice corresponding to “loose stool, or indigestion of food”. The behavioral changes of the mice were observed and the clinical manifestations of the “ aversion to cold and cold limbs” corresponding to the rectal temperature of the mice were detected. The clinical manifestations of “weight loss” corresponding to the body weight of mice were detected. The clinical manifestations of “listlessness and slow reaction” corresponding to the mental state and activity of the mice were observed (Li et al., 2022b).

### 2.6 Harvesting of organs and calculation of organ indices

After measuring the weight of the mice, they were euthanized using cervical dislocation performed on a sterile operating table. The spleen, thymus, and liver were excised with care, ensuring the removal of any attached surface fascia and adipose tissue. The organ index was determined via the following formula: Organ index = organ weight (mg)/body weight (g) (Liu et al., 2023).

### 2.7 Collection of blood samples and subsequent analysis

Following 12 hours of fasting and deprivation, mice from each group were selected for sampling. Blood samples were obtained by extracting them from the eyeball. After standing for 4 hours, the blood was centrifuged at 3000 rpm for 15 min to separate the serum. The serum levels of uric acid, alanine aminotransferase (ALT), aspartate aminotransferase (AST), and lactate dehydrogenase (LDH) were analyzed using an automated blood biochemistry analyzer.

### 2.8 Renal histopathological observation

Specific steps using HE staining to observe renal tissue included dissecting renal tissue, fixing with paraformaldehyde, dehydrating with ethanol, embedding in paraffin, sectioning, staining with hematoxylin and eosin, and microscopic observation. Detailed procedures can be found in the literature (Zhu et al., 2022).

### 2.9 Quantification of MDA and SOD levels in kidney tissue

The levels of MDA and SOD were measured using the thiobarbituric acid microplate method and the nitro-blue tetrazolium riboflavin microplate method, respectively. The specific techniques can be found in the referenced literature (Zhou et al., 2024b).

### 2.10 Determination of microbiota in intestinal contents

Under aseptic conditions, mice were euthanized via cervical dislocation, followed by dissection of the jejunum to the ileum segments and removal of intestinal contents using sterile forceps. The intestinal contents were then aliquoted into 50 mL sterilized centrifuge tubes containing glass beads, labeled, weighed, and stored at -20°C. Sterile water and the collected intestinal contents were introduced into the centrifuge tubes in a sterile environment. The tubes were placed in a temperature-controlled shaker and agitated for 30 min to ensure the complete release of microorganisms from the intestinal contents (Hui et al., 2020; He et al., 2019). The number of culturable microbial colonies in intestinal contents was determined by dilution plate counting. The plates inoculated with *E. coli* and bacteria were placed upside down into a 37°C constant temperature incubator for 24 hours of aerobic culture. The plates inoculated with *Bifidobacterium* and *Lactobacillus* were inverted and placed in an anaerobic incubator at 37°C for 24 hours. At the end of the culture, the plates were removed, and the number of colonies on each plate was counted by the manual counting method. Plates with colonies between 30 and 300 were selected to record data and were converted into colony-forming units per gram of sample (CFU/g) according to the dilution and inoculation volume (Wu et al., 2021).

### 2.11 Intestinal enzyme activity assay

Content sample collection and preparation were the same as section 3.10. After removing the intestinal contents from the jejunum to the ileum segment, the intestinal tract was longitudinally incised using sterile ophthalmic scissors. Residual intestinal contents were washed away with normal saline, and excess water on the intestinal wall tissue was removed using filter paper. Scrape the intestinal mucosa with a sterile coverslip, transfer it into a 50 mL sterile centrifuge tube containing glass beads, label and weigh it. Add sterile water in proportion and place it on a shaker for 30 min to obtain the crude enzyme solution. After oscillation, the crude enzyme solution was centrifuged at 3000 rpm for 10 min, and the supernatant was collected for further analysis (Qiao et al., 2023). The amylase, sucrase, and xylanase activities were measured using the DNS colorimetric method, while the protease activity was determined using the Folin-phenol method. For lactase activity, the ONPG method was employed. Enzyme activities in the supernatant were analyzed using a UV spectrophotometer and expressed as units per gram of intestinal contents or mucosa (U/g) (Qiao et al., 2022).

### 2.12 Intestinal microbial activity assessment

After obtaining the supernatant using the same method, microbial activity in the samples was detected using the FDA hydrolysis method. In each group, there were a total of four tubes: one blank tube and three sample tubes. A dry sterilized tube was used for the blank tube, and 2 mL of FDA reaction solution, 2 mL of acetone, and 10 µL of sample were added. The mixture was then incubated at 24°C for 90 min. In the sample tubes, also using dry sterilized tubes, 2 mL of the FDA reaction solution and 10 µL of the sample were added. The mixture was incubated at 24°C for 90 min. To terminate the reaction, 2 mL of acetone was added. The absorbance of the samples was then measured using a UV spectrophotometer at a wavelength of 490 nm (Yi et al., 2023).

### 2.13 Statistical analysis

SPSS 25.0 software was used for data processing and statistical analysis. The experimental data were first tested for normality and homogeneity of variance. If normal distribution and homogeneity of variance were satisfied, one-way analysis of variance was used for comparison between groups, and *post hoc* multiple comparisons were performed when necessary. If the data do not meet the assumptions of normal distribution or homogeneity of variance, the Kruskal-Wallis test is employed for statistical analysis. The significance level was set at α=0.05, and *P*<0.05 was considered statistically significant.

## 3 Results

### 3.1 Effect of Sishen Pill on immune organ indices in model mice

**Figure 1:**
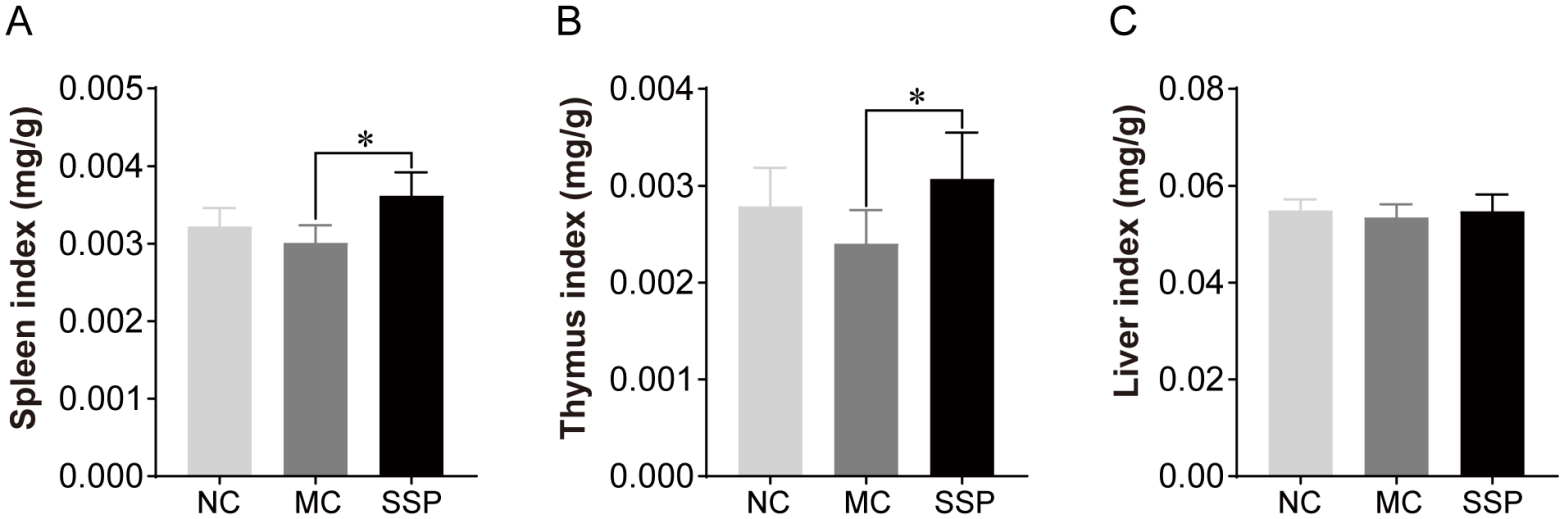
Organ indices were compared among the three groups. NC, blank control group; MC, spontaneous recovery group; SSP, Sishen Pill group; *P<0.05.

The organ index results, as illustrated in **Figure 1**, indicated that the spleen and thymus indices of the SSP group exhibited a noticeable increase in comparison to those of the MC group (*P*<0.05). However, no notable discrepancies were observed between the SSP and NC groups (*P*>0.05). Additionally, no discernible variances were observed in the liver index among the three groups (*P*>0.05).

### 3.2 Effect of Sishen Pill on blood biochemical indexes in model mice

**Figure 2:**
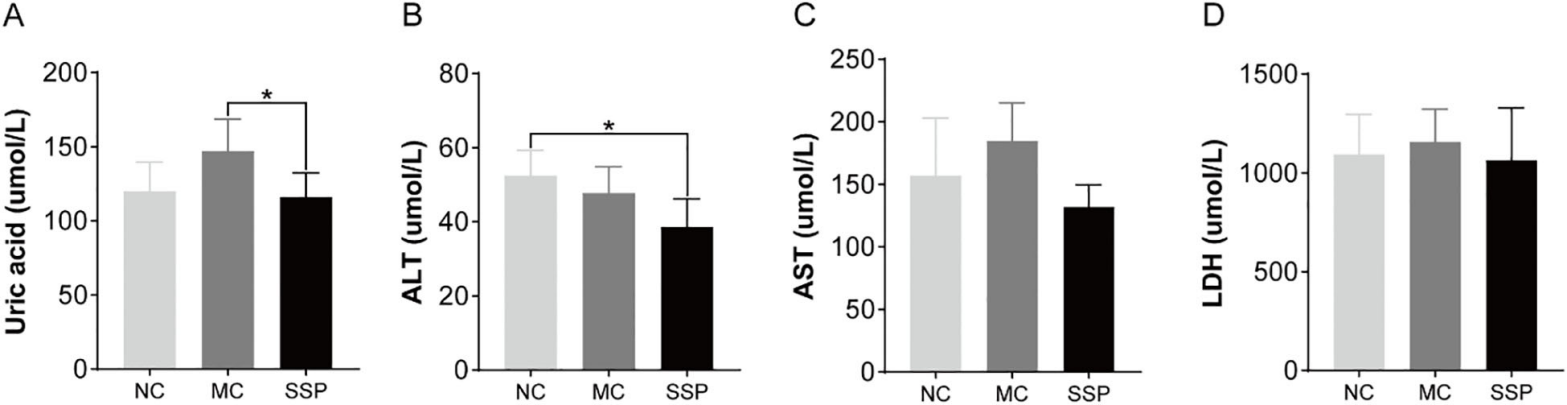
Blood biochemical indexes were compared among the three groups. **(A)** Uric acid. **(B)** Alanine aminotransferase (ALT). **(C)** Aspartate aminotransferase (AST). **(D)** Lactate dehydrogenase (LDH). NC, blank control group; MC, spontaneous recovery group; SSP, Sishen Pill group; *P<0.05.

According to the blood biochemical results, the uric acid content in the SSP group exhibited a marked reduction compared to the MC group (*P*<0.05). At the same time, no notable distinction was found between the SSP and NC groups (*P*>0.05; **Figure 2A**). The ALT content in the SSP group showed a significant decrease compared to that in the NC group. (*P*<0.05; **Figure 2B**). There were no discernible variances in the AST content and LDH activity among the three groups (*P*>0.05; **Figures 2C, D**).

### 3.3 Effect of Sishen Pill on kidney tissue in model mice

**Figure 3:**
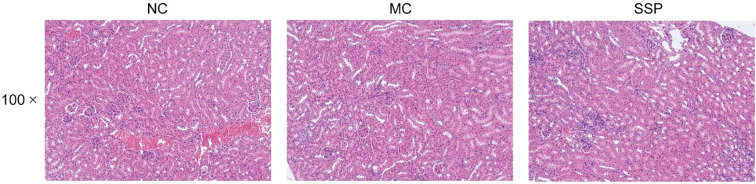
Renal histopathological observation. NC, blank control group; MC, spontaneous recovery group; SSP, Sishen Pill group.

**Figure 3** shows the morphological changes of the kidney in each group of mice. The kidney tissue morphology of mice in the NC group was expected. The glomeruli were densely distributed, the structure was complete and precise, most were round or oval, and the renal cysts and tubules were regular without apparent dilatation. Mice in the MC and SSP groups had normal kidney tissue morphology, and no abnormal pathological changes were observed.

### 3.4 Effect of Sishen Pill on MDA level and SOD activity in model mice

**Figure 4:**
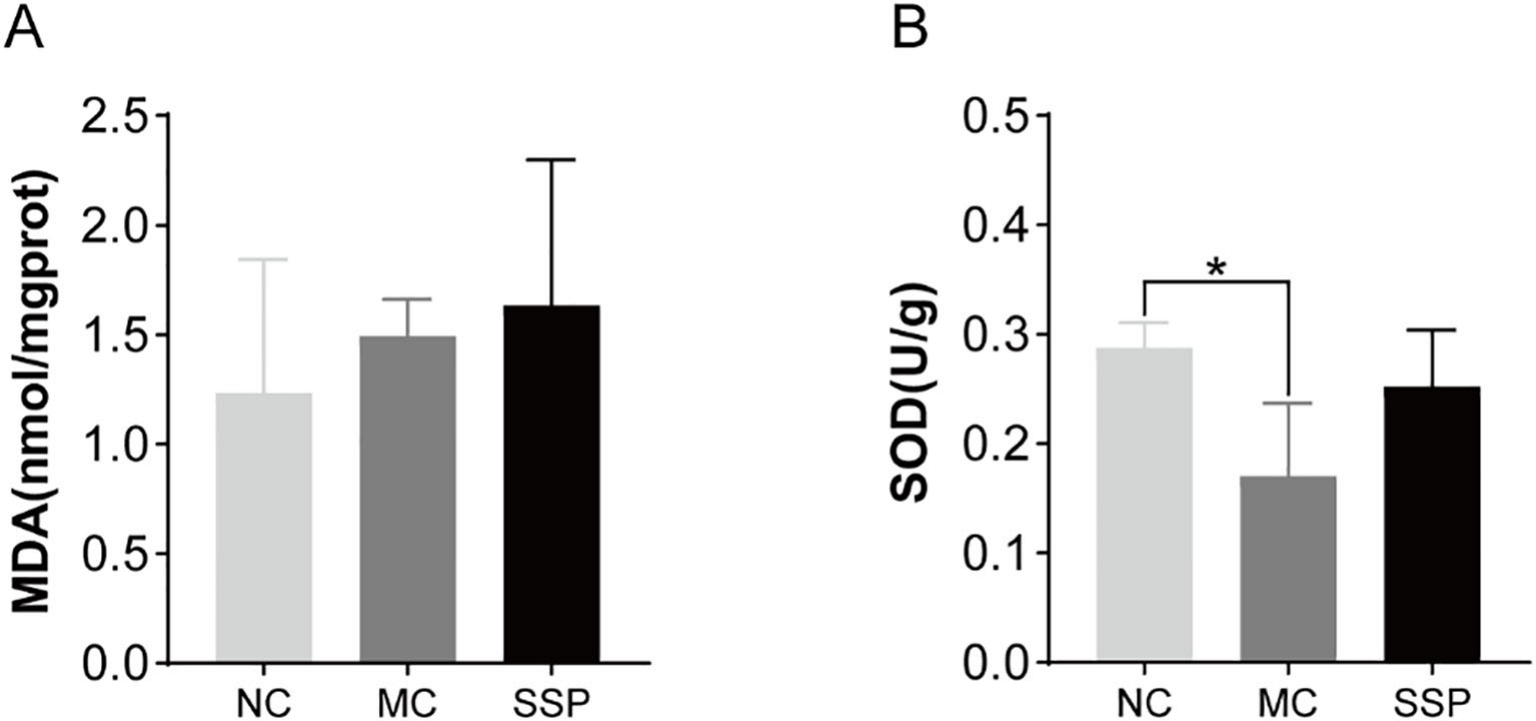
Comparison of MDA levels and SOD activity among the three groups. **(A)** Malondialdehyde (MDA). **(B)** Superoxide dismutase (SOD). NC, blank control group; MC, spontaneous recovery group; SSP, Sishen Pill group; *P<0.05.

In **Figure 4**, no notable disparities were observed in the MDA level of the kidneys among the three groups of mice (*P*>0.05). In comparison to the NC group, the MC group showed significantly lower SOD (superoxide dismutase) activity in the kidneys (*P*<0.05). However, there were negligible variances observed in SOD activity between the SSP and NC groups (*P*>0.05). After administration of decoction of SSP, the activity of SOD in the kidney of mice with diarrhea with KYDS has an upward trend, indicating that SSP helps improve the ability of the body to remove oxygen-free radicals.

### 3.5 Effect of Sishen Pill on intestinal microorganisms in model mice

**Figure 5:**
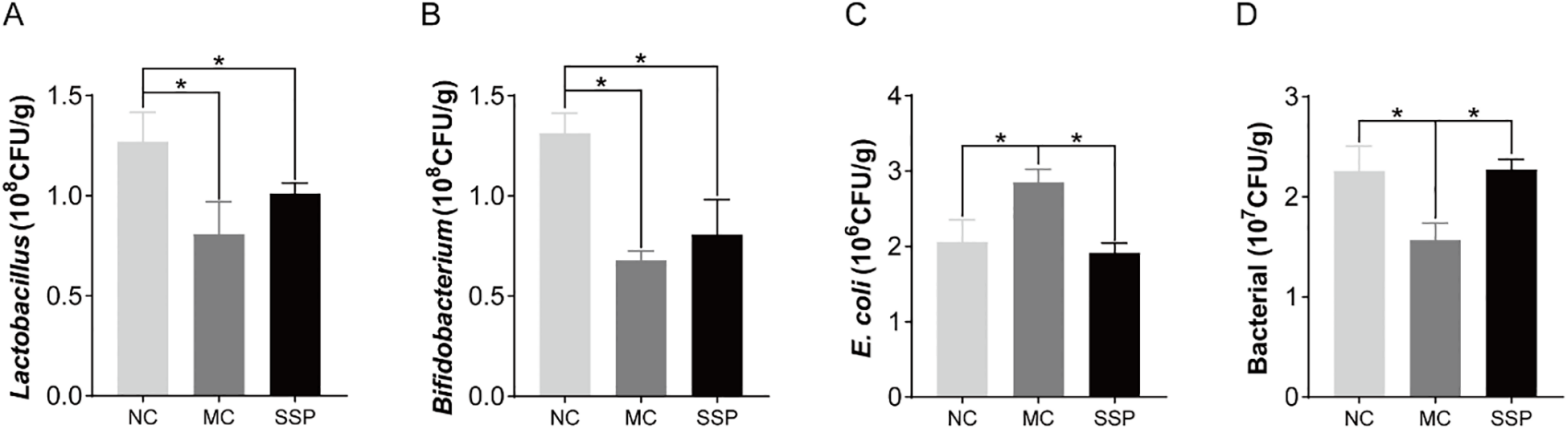
Comparison of the number of intestinal microbial colonies among the three groups. **(A)** *Lactobacillus*. **(B)** *Bifidobacterium*. **(C)** *E. coli*. **(D)** Bacterial. NC, blank control group; MC, spontaneous recovery group; SSP, Sishen Pill group; *P<0.05.

The number of *Lactobacillus* and *Bifidobacterium* colonies in the intestinal contents of the MC and SSP groups was substantially less than that of the NC group (*P*<0.05). Compared with the MC group, the number of colonies of two beneficial bacteria in the SSP group did not increase significantly but only showed an upward trend (*P*>0.05; **Figures 5A, B**). The number of *E. coli* colonies in the intestinal contents of mice in the MC group was significantly higher than that in the NC and SSP groups (*P*<0.05; **Figure 5C**). The number of total bacterial colonies in the intestinal contents of the MC group was significantly lower than that of the NC and SSP groups (*P*<0.05; **Figure 5D**). These results indicate that the gut microbiota after treatment with the SSP has changed, mainly reflected in the fact that the water decoction of SSP reduces the number of *E. coli* colonies in the unit intestinal contents of mice and increases the number of total bacterial colonies in the unit intestinal contents. However, the effect of the SSP on the growth of beneficial bacteria *Lactobacillus* and *Bifidobacterium* in the gut of mice was not evident in this study.

### 3.6 Effect of Sishen Pill on intestinal enzyme activities in model mice

**Figure 6:**
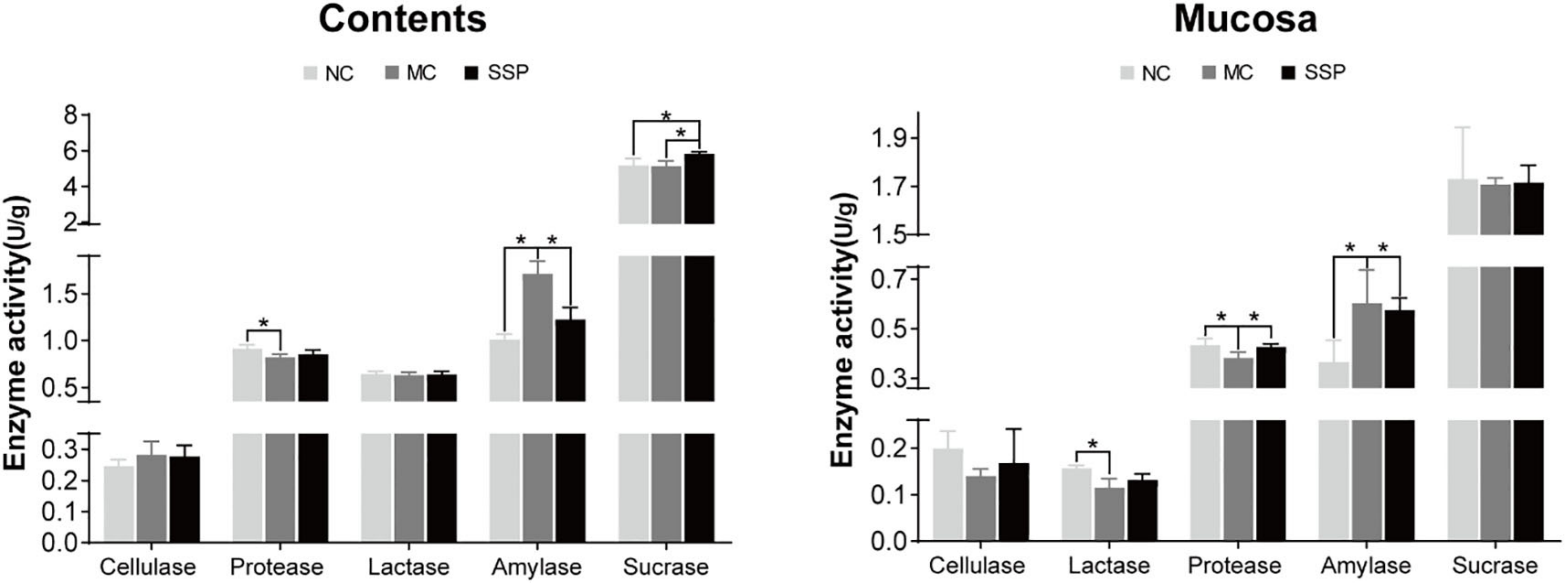
Comparison of enzyme activities. **(A)** Changes in enzyme activities in the intestinal contents of mice in each group. **(B)** Changes in enzyme activities in the intestinal mucosa of mice in each group. NC, blank control group; MC, spontaneous recovery group; SSP, Sishen Pill group; *P <0.05.

As shown in **Figure 6**, the amylase activities in mice’s intestinal contents and mucosa in the NC and SSP groups exhibited a significant reduction compared to those in the MC group (*P*<0.05). The protease activities in the intestinal mucosa of mice in the NC and SSP groups showed a significant increase compared to those in the MC group (*P*<0.05). There were no significant differences between the NC and SSP groups (P > 0.05). The protease activities in intestinal contents and lactase activities in the mucosa of the MC group were significantly lower than those of the NC group (*P*<0.05). In contrast, those of the SSP group showed an upward trend (*P*>0.05). The sucrase activity in the intestinal contents of the SSP group was significantly higher than that of the NC and MC groups (*P*<0.05). The three groups had negligible variances in lactase and cellulase in intestinal contents and sucrase and cellulase in mucosa (*P*>0.05). According to the above results, we can see that SSP has a significantly better improvement effect on the activity of amylase in the intestine and the activity of protease in the intestinal mucosa than the natural recovery group and substantially increases the activity of sucrase in the intestinal contents, and tends to restore the activity of protease in the intestinal contents and lactase in the mucosa.

### 3.7 Effect of Sishen Pill on intestinal microbial activity in model mice

**Figure 7:**
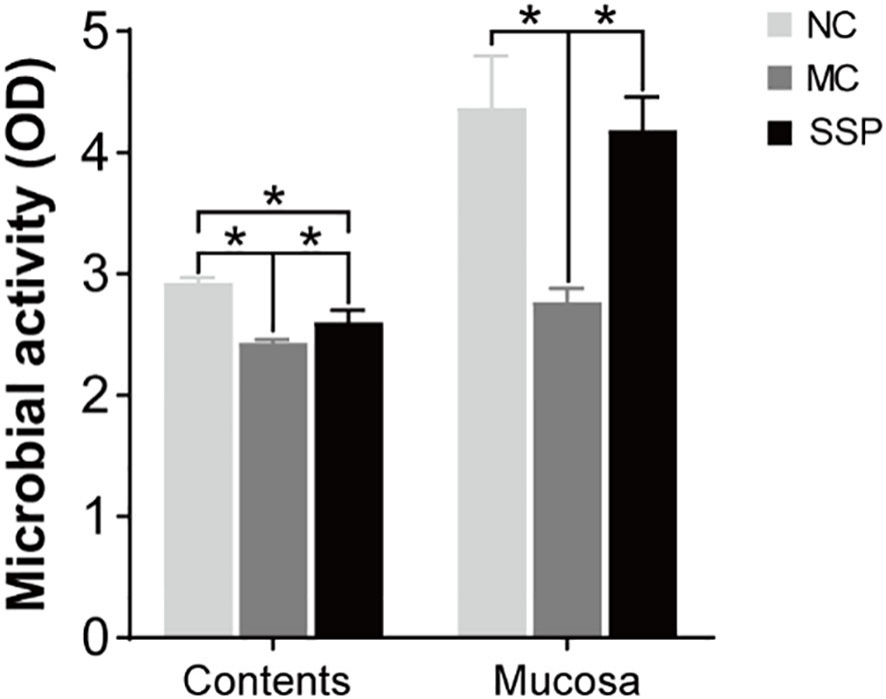
Comparison of intestinal contents and mucosa microbial activity. NC, blank control group; MC, spontaneous recovery group; SSP, Sishen Pill group; *P<0.05.

The results of microbial activity showed that the intestinal microbial activity of mice in the MC group was significantly lower than that in the NC and SSP groups in intestinal contents and intestinal mucosa. The microbial activity in the intestinal contents of mice in the SSP group was significantly lower than that in the NC group (*P*<0.05; **Figure 7**). The results suggested that SSP could dramatically improve the microbial activity in the intestinal mucosa. Although the microbial activity in the intestinal contents was still different from that in the NC group, it was higher than that in the MC group without SSP treatment, indicating that the improvement of the microbial activity in the intestinal mucosa was better than that in the intestinal contents.

### 3.8 Correlation between blood biochemical indexes and the number of intestinal microbial colonies

**Figure 8:**
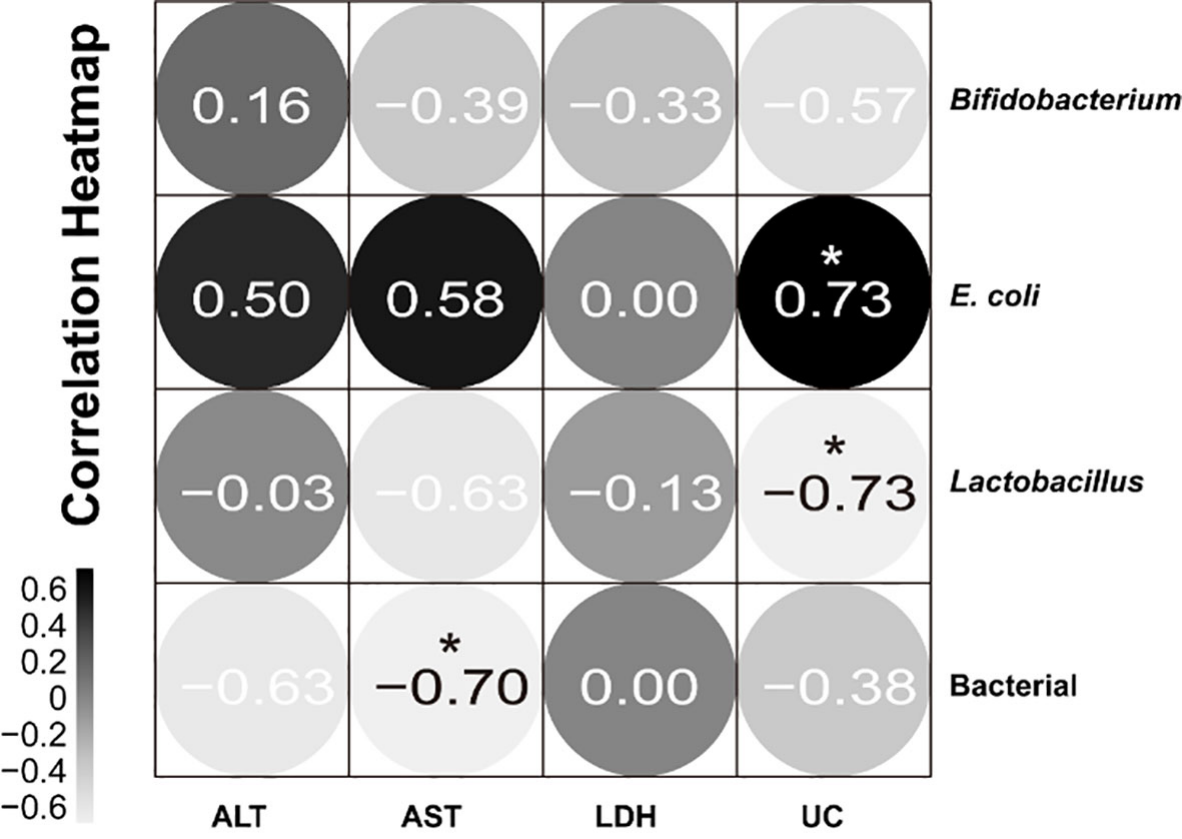
Correlation between blood biochemical indexes and the number of intestinal microbial colonies. Black indicates positive correlation and gray indicates negative correlation; the closer the correlation coefficient is to 1, the greater the correlation between the two factors, and the closer the correlation coefficient is to 0, the more independent the two factors are. *P<0.05.

We performed Pearson correlation studies between blood biochemical measures and gut microbiota. Gut microbiota affects the function of multiple systems, so the correlation between them was analyzed. Gut microbiota affects the function of various systems, so the correlation between them was analyzed. **Figure 8** shows that the serum uric acid level is significantly positively correlated with the colony count of *E. coli*, and significantly negatively correlated with the colony count of *Lactobacillus*. The total bacterial colony count is significantly negatively correlated with AST. It can be seen that changes in serum uric acid and AST levels are associated with changes in the colony counts of *E. coli*, *Lactobacillus*, and total bacteria.

## 4 Discussion

### 4.1 The regulatory effects of Sishen Pill on intestinal microorganisms and enzyme activity

This study found that the administration of SSP in mice with diarrhea with KYDS could reduce the number of *E. coli* colonies in intestinal contents, decrease intestinal amylase activity, increase protease activity in the intestinal mucosa, and enhance overall intestinal microbial activity. Evidence positions gut microbiota dysbiosis as a pivotal driver of diarrhea pathophysiology, where taxonomic and functional microbial imbalances directly contribute to disease onset, severity trajectories, and therapeutic responsiveness. Diarrheagenic pathogens, including facultative anaerobic Gram-negative bacteria (*E. coli*, *Salmonella*, *Shigella*, *Vibrio cholerae*) and Gram-positive Clostridium difficile, employ dual pathogenic mechanisms: (1) direct cytotoxicity via enterotoxin production, which disrupts intestinal epithelial tight junctions and ion transport, and (2) ecological disruption of commensal microbiota through competitive exclusion or antimicrobial peptide secretion, ultimately reducing the number of microbial colonies and altering community structure (Li et al., 2021). Antibiotic treatment induced intestinal overgrowth of opportunistic pathogens and altered gut microbiota composition, resulting in functional dysbiosis of gut microbiota (Shao et al., 2020). SSP can effectively alleviate dextran sodium sulfate (DSS)-induced colitis by regulating the interaction between inflammatory dendritic cells and gut microbiota (Chen et al., 2020). For colitis with Yang deficiency of the spleen and kidney syndrome, SSP can improve clinical symptoms by improving the composition of gut microbiota, and regulating some characteristic microbiota, regulating the balance of pro-inflammatory factors and anti-inflammatory factors in colon tissue, and inhibiting the differentiation of inflammatory follicular dendritic cells (Ge et al., 2022). In addition, SSP alleviates diarrhea by improving the structure and diversity of the intestinal mucosal microbial community and improving energy metabolism by affecting Na^+^-K^+^-ATPase and Ca^2+^-Mg^2+^-ATPase (Zhu et al., 2023). Notably, experimental evidence from colitis models reveals that SSP treatment modulates the Rho/ROCK signaling pathway, enhances tight junction protein expression, preserves colonic mucosal barrier integrity, and prevents pathogenic infiltration, thereby ameliorating 2,4,6-trinitrobenzene sulfonic acid-induced chronic colitis (Zhang et al., 2021). In the previous research, the active components of SSP were identified using ultra-high-performance liquid chromatography. By comparing with standard products, seven components were detected, including phenylpropanoids, alkaloids, flavonoids, coumarins and their derivatives, terpenoids, and other components (Li et al., 2024). Studies have found that phenylpropanoids (such as pimpinellin) can increase the levels of beneficial intestinal probiotics (S24–7 and Lactobacillaceae) while reducing the presence of harmful bacteria (Enterobacteriaceae) (Lv et al., 2025). A large number of studies have shown that flavonoids exert anti-cancer effects by reshaping the gut microbiota, which can convert flavonoids into bioactive metabolites with anti-cancer activity (Wang et al., 2022a). The inhibitory effect of SSP on *E. coli* colonization aligns with prior findings, suggesting that its therapeutic effect on diarrhea with KYDS may involve the suppression of *E. coli* proliferation, attenuation of enterotoxin-induced epithelial toxicity, and restoration of commensal microbiota equilibrium. Recent research has found that the 75% SSP combined with 60 mg/kg sodium propionate significantly alleviated symptoms related to diarrhea with KYDS, and it enhanced intestinal immune function and reduced intestinal inflammation by modulating the gut microbiota (Guo et al., 2025).

Amylase and protease are produced by the gut or gut microbiota and are closely related to the digestive and absorptive functions of the small intestine (Wu et al., 2021). Enzyme activity is tightly linked to energy metabolism: digestive enzymes (such as amylase, lactase, and protease) act as key drivers of energy metabolism by hydrolyzing macromolecular nutrients into absorbable small molecules (e.g., glucose, galactose, and amino acids) that serve as core substrates for energy production. Changes in enzyme activity directly affect the efficiency of nutrient digestion and absorption, thereby regulating energy supply and homeostasis, and modulating energy metabolism by altering the production of energy substrates, which in turn support various physiological functions dependent on energy supply (Di et al., 2025). Previous studies observed significantly elevated intestinal amylase activity in *Foliuem sennae-induced* spleen deficiency diarrhea mice, potentially reflecting a compensatory mechanism to counteract impaired energy supply (Hu et al., 2018). Subsequent experiments evaluating adenine-*Foliuem sennae* co-administration revealed elevated sucrase activity and depressed lactase activity in the intestinal contents of mice treated with adenine (25/50 mg/(kg·d) for 14 days plus *Foliuem sennae* (10 g/day for 7 days) (Li et al., 2023b). Chronic consumption of vegetable oil or lard reduces protease activity, correlating with decreased abundance of enzyme-producing gut microorganisms (Qiao et al., 2022). In contrast, Baohe Pill enhances intestinal protease activity in mice with diarrhea induced by a high-fat/high-protein diet (Guo et al., 2022). Our study demonstrates that while amylase activity remained elevated in model group mice, SSP treatment normalized amylase activity to that of healthy controls, and increased mucosal protease activity, suggesting its dual role in restoring energy homeostasis and enhancing protein digestive capacity. Notably, sucrase activity was significantly higher in the intestinal contents of the SSP group than in the NC and MC groups. These findings suggest a potential mechanism by which SSP enhances sucrase activity, warranting further investigation. Intestinal enzyme activity is closely associated with diarrhea. Congenital lactase deficiency impairs intestinal lactase function, preventing lactose digestion and leading to osmotic diarrhea (Wanes et al., 2019). Similarly, sucrase-isomaltase deficiency, another congenital disorder, results from the loss of the invertase complex at the small intestinal brush border, causing impaired carbohydrate absorption. Undigested disaccharides entering the colon are fermented by gut microbiota, producing metabolites such as short-chain fatty acids and hydrogen, which induce osmotic fermentative diarrhea. Clinically, this manifests as watery stools, abdominal pain, distension, and cramps (Smith et al., 2021). Furthermore, numerous studies on diarrhea have documented altered activities of lactase, sucrase, amylase, and other enzymes, emphasizing that restoring enzyme activity alleviates diarrheal symptoms (Shu et al., 2021; Guo et al., 2019; Hui et al., 2018; Wu et al., 2020). Enzymes derived from the host or microbiota play a crucial role in regulating the gut microbiota by participating in biochemical reactions, influencing it from multiple dimensions. On one hand, enzymatic activities directly modulate the composition and overall balance of the microbial community. On the other hand, they alter the intestinal environment, thereby shaping the microbial ecosystem (Jiang et al., 2024). Studies have shown that the decreased activities of proteases, xylanases, amylases, etc., are primarily attributed to the reduction in the number of gut microbes producing these enzymes. The improvement in enzymatic activity may reflect the optimization of the quantity or function of enzyme-producing microbes (Qiao et al., 2022). Certain enzymes assist in metabolizing specific types of sugars, such as lactose and xylan, or are involved in the production of short-chain fatty acids, which are crucial for maintaining intestinal health (Turnbaugh et al., 2006). Enzymes act as a hub in the balance of gut microecology by regulating the microbial community and its metabolic environment. The dynamic correlation between enzymatic activity and the number of enzyme-producing microbes, together with the impact of carbohydrate metabolism on SCFA production, jointly constitute an “enzyme-microbe-metabolite” interaction network, providing an important perspective for revealing the regulatory mechanisms of intestinal health.

The FDA hydrolysis method was initially used to measure microbial activity in soil and straw litter and was gradually used to measure microbial activity in various substances (Frlolov et al., 2022). FDA can be hydrolyzed by a variety of enzymes, such as proteases, lipases, and esterases, and the enzyme activity measured by FDA hydrolysis is usually related to other factors such as biomass, ATP content, oxygen consumption, or optical density, so it is commonly expressed as total enzyme activity, which is a widely used method for measuring total enzyme activity (Dzionek et al., 2018; Schnurer and Rosswall, 1982). In this experiment, the FDA hydrolysis method was used to detect the overall metabolism of microorganisms in intestinal contents and mucosa. The increase in microbial activity in this experiment indicates the positive effect of SSP on overall intestinal enzyme activity.

Therefore, we speculated that SSP may improve diarrhea with KYDS by reducing the number of *E. coli*, regulating the activities of intestinal amylase, protease, and sucrase, and increasing microbial activity. This study employed traditional microbial culture techniques. Although this method has limitations, its results in the current research still provide important clues for revealing the changes in microbial communities after the intervention of Sishen Pill. In follow-up in-depth research, we will apply more advanced technologies such as 16S rRNA gene sequencing or metagenomics to more comprehensively explore microbial diversity and composition, thereby deeply revealing the microecological mechanism by which Sishen Pill improves diarrhea in kidney yang deficiency syndrome.

### 4.2 Sishen Pill improves oxidative stress and metabolic function in model mice

The organ index is commonly used to assess the overall state of immune function, and the changes in the organ index can reflect the effects of food and drugs on organs, providing reference data for animal experiments (Li et al., 2023a). Previous studies have shown that gavage of 50 mg/(kg·d) adenine suspension for 14 days combined with gavage of 10 g/(kg·d) *Foliuem sennae* decoction for 7 days results in a decrease in the thymus index and an increase in the spleen index (Li et al., 2023b). In this experiment, after the same method was used to establish the model, diarrhea with KYDS mice was treated with SSP for one week, and the changes in the thymus, spleen, and liver indices were observed. The results showed that the thymus and spleen indices of SSP-treated mice recovered, which was significantly different from the MC group. This suggests that SSP has a specific effect on improving the immune function of mice with KYDS. Still, the organ index can only roughly observe the immune function, which needs to be further observed combined with other indicators.

The liver and kidneys are critical detoxification and excretory organs, rendering their potential injury a critical consideration in drug safety evaluation. Approximately one-third of uric acid produced in the human body is excreted via the intestine, with the gut microbiota playing a pivotal role in this excretory process. Emerging evidence has highlighted distinct microbial associations with uric acid metabolism: in diabetic patients under 55 years of age, uric acid levels are positively correlated with *Escherichia*-*Shigella*, whereas an inverse relationship is observed with *Faecalibacterium* amounts (Zhang et al., 2022). This finding aligns with earlier research demonstrating that *Escherichia*-*Shigella* secretes xanthine deaminase, an enzyme that catalyzes the conversion of hypoxanthine and xanthine to uric acid (Xi et al., 2000). Building on these mechanistic insights, recent studies have shown that plant-derived natural products can target gut microbiota to modulate uric acid homeostasis through dual pathways: promoting purine degradation to reduce uric acid biosynthesis and enhancing renal excretion (Dong et al., 2025). Concurrently, the gut microbiota exerts protective effects against hyperuricemia-induced renal injury via anti-inflammatory and antioxidant mechanisms. A notable example is *Lacticaseibacillus paracasei* CPU202306, which lowers serum uric acid through a multifaceted approach: direct degradation of uric acid, inhibition of hepatic xanthine oxidase and adenosine deaminase, stimulation of short-chain fatty acid production in the colon to regulate uric acid transporters, and modulation of gut barrier function by enhancing tight junction proteins. Additionally, this probiotic strain reshapes the microbiota toward a healthier composition, reduces pathogenic bacteria, inhibits renal inflammatory signaling, and maintains renal health through regulation of amino acid metabolic pathways associated with the gut-kidney axis (Zhou et al., 2025). The influence of gut microbiota and their metabolites has significant effects on the gut-kidney axis (Yu et al., 2025). Based on this background, Trimethylamine N-oxide (TMAO), a nitrogenous uremic toxin generated by gut bacterial degradation of choline, exacerbates renal fibrosis, impairs renal function, and accelerates chronic kidney disease progression. Notably, dysfunction of this bidirectional axis creates a vicious cycle, as renal injury further elevates TMAO levels (Xie et al., 2024a). This pathogenic mechanism underscores the therapeutic potential of microbiota modulation, as exemplified by Sishen Pill, which alleviates diarrhea with KYDS by reducing TMAO-mediated inflammatory responses transmitted via the gut-kidney axis (Xie et al., 2024b). Consistent with previous findings, the present study confirms a positive correlation between serum uric acid and *E. coli* through correlation analysis, alongside an inverse correlation with *Lactobacillus*. These results suggest a potential mechanism through which SSP may reduce uric acid levels through reduction of *E. coli* counts and increase the number of *Lactobacillus*, thereby reducing the damage to the kidneys, offering a potential scientific basis for translational applications in disease therapy. To further validate the direct effects of the SSP on the gut-kidney axis, future research should explore kidney gene expression and the interaction between the microbiota and immunity.

It has been found that gut microbiota is correlated with serum liver damage indicators ALT and AST. *Enterococcus* is positively correlated with ALT and AST (Tan et al., 2023). The overgrowth and translocation of *Enterococcus* to the liver activate the pathogen recognition system and the production of inflammatory factors, triggering the liver’s inflammatory response and causing liver damage (Llorente et al., 2017). Another study found that *Aercoccus*, *Oscillospira*, and *Prevotella* were negatively correlated with AST after 14 days of gavage with 50 mg/(kg·d) adenine suspension, further suggesting a link between characteristic bacterial genera of mouse intestinal mucosa and liver function (Fang et al., 2025). In this experiment, AST level was negatively correlated with the total number of bacteria, again emphasizing the mutual relationship between liver injury and gut microbiota. In conclusion, the gut microbiota may serve as a key link between uric acid metabolism and liver injury, and SSP-targeted regulation of gut microbiota may become a new strategy for the treatment of metabolic syndrome (hyperuricemia, liver injury, etc.), but further mechanism research is needed to support it.

LDH, a cytoplasmic enzyme critical for glycolysis, converts lactate to pyruvate in aerobic metabolism to fuel the citric acid cycle and ATP production, its activity level reflects tissue oxygenation and energy metabolism status, with impaired glycolysis leading to reduced ATP synthesis substrates and abnormal energy metabolism (Wei et al., 2022; Li et al., 2015; Gan et al., 2016; Deng et al., 2017). The increased serum LDH level is an essential reflection of tissue and cell damage (Gan et al., 2016). Yang deficiency state is closely related to energy metabolism, and the decrease of LDH indicates abnormal energy metabolism, and the energy supply of sugar oxidation is inhibited. Therefore, the animal model of Yang deficiency has symptoms such as decreased body temperature and fear of cold (Xue et al., 2001). As a critical formula for treating diarrhea with KYDS, SSP is also widely used in various intestinal diseases. Studies have found that SSP can improve the activity of LDH in the colon of rats with colitis and increase energy supply by interfering with the energy metabolism of intestinal epithelial cells to ensure the integrity of intestinal epithelial cells’ structure and function, thereby reducing the damage of inflammatory substances and alleviating intestinal inflammation (Wang et al., 2019). The results of this experiment revealed minimal variations in serum LDH activity across the different groups of mice. This suggests that serum LDH stability might obscure the metabolic changes occurring in local tissues. Therefore, the therapeutic effect of SSP is more likely to be mediated through its impact on local intestinal energy metabolism. To gain a more comprehensive understanding of SSP’s regulation of energy metabolism, future studies should include tissue-level LDH measurements (such as in the intestine and liver), as reliance solely on serum markers may lead to false-negative conclusions. Moreover, SSP’s regulatory effect on the energy metabolism of intestinal epithelial cells offers potential therapeutic insights for inflammatory bowel diseases, such as colitis, particularly in patients with Yang deficiency and associated energy metabolism disorders.

Next, we measured the MDA level and SOD activity in kidney tissue to assess the renal oxidative stress in each group of mice. MDA is a lipid peroxide formed by many free radicals generated when the body’s oxidative and antioxidant systems are out of balance. Oxygen free radicals attack the polyunsaturated fatty acids of biological membranes, increasing the content of MDA, which indirectly reflects the extent of free radical-induced cellular damage (Lin and Chen, 2000). SOD catalyzes the dismutation of superoxide radicals into oxygen and hydrogen peroxide with high specificity and efficiency and is the only known enzyme that can directly scavenge free radicals (Borgstahl and Oberley-Deegan, 2018). The level of renal SOD is an important index reflecting the repair ability of the kidney oxidative stress injury. According to the results of previous experiments, when MDA increases and SOD decreases, it indicates that the accumulation level of free radicals in the body has exceeded the body’s ability to remove them. When MDA decreases and SOD increases, it means that the accumulation level of free radicals in the body decreases, and the body’s ability to remove free radicals recovers. Oxygen-free radical disorder was improved (Zhu et al., 2001; Lu et al., 2023). In this experiment, SOD activity in the MC group was significantly lower than that in the NC group, indicating that SSP may mitigate renal oxidative damage by enhancing SOD expression. These results, combined with the observed correlations between specific gut microbiota (e.g., *Akkermansia*, *UCG-005*, *Lachnospiraceae NK4A136 group*, *Lactococcus*, and *Butymonas*) and oxidative stress markers (Liu et al., 2024), support the hypothesis that SSP may alleviate renal oxidative damage by modulating SOD activity and gut microbial composition. Further studies are warranted to elucidate the mechanistic role of these microbes in SSP-mediated renal protection and explore their potential as therapeutic targets for oxidative stress-related kidney disorders.

## 5 Conclusion

In summary, our findings suggest that SSP can modulate gut microbiota, manifested as inhibition of pathogenic *E. coli* (positively correlated with uric acid), regulation of enzyme activities, including normalization of amylase activity, enhancement of sucrase/protease function, and enhancement of intestinal microbial activity; The thymus/spleen index and renal oxidative stress (SOD up-regulation) were improved. However, a limitation of this study is that a causal relationship between microbiota and metabolites was not established. Future studies should employ sterile models and microbial transplantation to establish causal relationships, combine multi-omics to map host-microbe networks, and conduct staged clinical trials to evaluate the translational potential of SSP in microbiologically associated metabolic diseases. This work positions SSP as a promising multisystem regulator of microbial-enzyme-metabolism interactions that requires mechanistic and clinical validation.

## Data Availability

Publicly available datasets were analyzed in this study. This data can be found here: The datasets used and analyzed during the current study are available from the corresponding author upon reasonable request.
